# ﻿Scleractinian coral (Cnidaria, Hexacorallia, Scleractinia) diversity of the Mersing Islands, Peninsular Malaysia

**DOI:** 10.3897/zookeys.1102.82228

**Published:** 2022-05-20

**Authors:** Jen Nie Lee, Kee Alfian Abd Adzis, Lutfi Afiq-Rosli, Jani T. I. Tanzil, Albert Apollo Chan, Md Nizam Ismail, Khodzori Fikri Akmal, Yang Amri Affendi

**Affiliations:** 1 Faculty of Science and Marine Environment, Universiti Malaysia Terengganu, 21030 Kuala Nerus, Terengganu, Malaysia; 2 Marine Ecosystem Research Centre, Faculty of Science and Technology, Universiti Kebangsaan Malaysia, 43600 UKM Bangi, Selangor, Malaysia; 3 Department of Biological Sciences, National University of Singapore, 16 Science Drive 4, Block S3 Level 4, 117558, Singapore, Singapore; 4 Tropical Marine Science Institute, National University of Singapore, 119227 Singapore, Singapore; 5 Division of Marine Park and Resources Management, Department of Fisheries, 62628 Putrajaya, Malaysia; 6 Fisheries Research Institute, 11960 Batu Maung, Penang, Malaysia; 7 Borneo Marine Research Institute, Universiti Malaysia Sabah, Jalan UMS, 88400, Kota Kinabalu, Sabah, Malaysia; 8 Institute of Ocean and Earth Sciences, Universiti Malaya, 50603 Kuala Lumpur, Malaysia

**Keywords:** Biodiversity, conservation, hard coral, Johor, marine protected area, South China Sea

## Abstract

We present a comprehensive checklist of scleractinian (hard) corals for the Mersing Islands, Malaysia based on surveys conducted at 24 reefs across protected and unprotected marine areas. A total of 261 species of corals from 16 families and one *incertae sedis* (*Pachyseris* spp.) were recorded, along with ten records that are new for the east coast of Peninsular Malaysia. Compared against the IUCN Red List, 46.7% of coral species found in the Mersing Islands were of Least Concern (LC), 29.5% as Near Threatened (NT) and 16.4% Vulnerable (V). Only one recorded species, *Pectiniamaxima* (Moll & Best, 1984), was listed as Endangered (EN). Baseline species diversity data are essential for the monitoring and management of marine biodiversity, especially within marine protected areas. With both protected and unprotected coral reef areas in the vicinity of the widely scattered Mersing Islands, the diversity and distribution of coral species can be used as the basis for area-based conservation and management strategies. The diversity and abundance of scleractinian corals of each island or area should be surveyed periodically to ensure the appropriate level of protection is afforded to retain scleractinian biodiversity in this region.

## ﻿Introduction

Scleractinian corals, commonly referred to as hard corals, are a group of animals belonging to the order Scleractinia under the Phylum Cnidaria. These organisms are the backbone of coral reefs, which support high species diversity, provide goods and services (e.g., food, coastal protection, tourism), and provide substantive support to people worldwide (Praveena et al. 2012; Huang et al. 2016; Hoegh-Guldberg et al. 2019). Coral reefs in Malaysia are estimated to cover about 4,006 km^2^ (Praveena et al. 2012), with most reefs found in Sabah and along the east coast of Peninsular Malaysia, and in limited areas in Sarawak and the west coast of Peninsular Malaysia (UNEP 2007). A total of 398 species of scleractinian corals (Huang et al. 2015) and 925 species of reef fishes (Chong et al. 2010) have thus far been recorded from the shallow fringing reefs along the coasts of Peninsular Malaysia alone. These reefs are located at Sunda Shelf, within and near the western edge of the Coral Triangle, a marine biodiversity hotspot that is home to 627 species of zooxanthellate corals (Veron et al. 2015).

Malaysia, as a megadiverse country, is dedicated to fulfilling the Convention on Biological Diversity (CDB) agreement (Tong 2020). With the launch of the National Policy on Biological Diversity in 2016, Malaysia aims to further safeguard both key terrestrial and marine ecosystems, as well as species and genetic diversity (Goal 3) (Ministry of Natural Resources and Environment 2016). Knowledge and data on the biodiversity of Malaysia’s vast marine areas will therefore be crucial for stakeholders and policy makers to identify suitable areas for ecological protection. To date, studies that have reported on the reef-building coral biodiversity around Peninsular Malaysia are somewhat limited. A review by Affendi and Rosman (2011) found only six research articles on scleractinian diversity published for the coral reef-rich areas along the east coast of Peninsular Malaysia, most of which were based on surveys conducted only around highly visited tourist islands that are under the jurisdiction of the Department of Fisheries Malaysia (DOF), e.g., Pulau Redang and Pulau Tioman (e.g., Toda et al. 2007; Akmal et al. 2019).

The Mersing Islands comprise one of the largest archipelagos off the east coast of Peninsular Malaysia. With 58 islands (Said et al. 2021), this region is not only known for its coral reefs but also for its seagrass ecosystems (Ooi et al. 2011; Ponnampalam et al. 2015). Geologically, the Mersing Islands originated ~350 million years ago, and they are currently evaluated as a potential National Geopark for their unique geological and cultural heritage (Said et al. 2021). This elevated status will not only affect the islands but also the surrounding marine life, both in terms of increased protection and increased tourism. Biodiversity data in the area will therefore be extremely valuable to advise any development and/or management plans for the Mersing Islands. A sole report that recorded 155 species of scleractinian corals from four islands (Pulau Dayang, Pulau Pemanggil, Pulau Tinggi and Batu Tikus) (Harborne et al. 2000) was the main literature source for coral biodiversity in the Mersing Islands prior to this study, aims to provide a comprehensive updated species checklist of scleractinian corals for the coral reefs around the Mersing Islands.

## ﻿Methods

**Figure 1.**
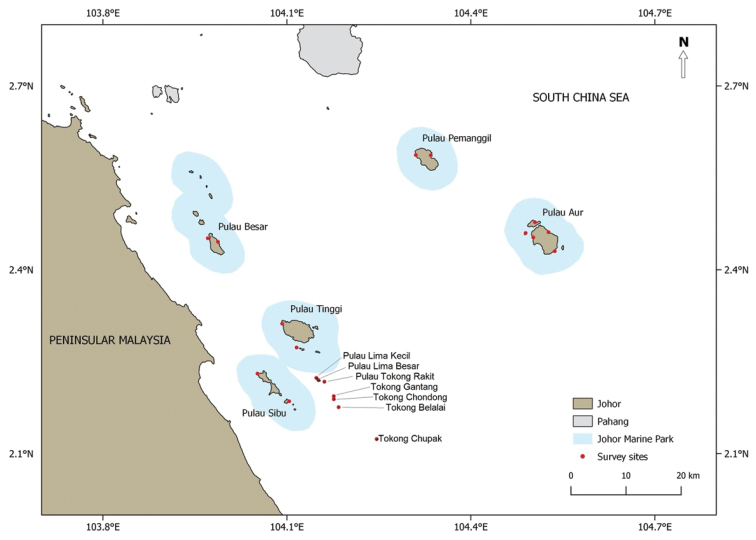
Survey areas at the Mersing Islands. Johor Marine Park protected area are two nautical miles away from the low tide shoreline of each gazetted island

The study area comprised islands on the east coast of Johor, Peninsular Malaysia, referred to as the Mersing Islands. Underwater surveys were carried out during two expeditions, one in 2012 (“Marine Park Biodiversity Expedition)” and one in 2016 (“Johor 8 Islands Expedition”). Parts of the Mersing Islands (Fig. 1) are protected under the unique overlapping protection by both Malaysia’s Federal (known as Johor Marine Park) and Johor State jurisdictions, i.e., these reefs are protected under the Fisheries Act of 1985 (Federal) as well as by the Johor State government, following the establishment of the Johor National Park in 1990. Both authorities promote the protection, preservation and management of the natural breeding ground and habitat of aquatic life. In 2013, the protected area that falls within Mersing Islands was renamed ‘Sultan Iskandar Marine Park’, and entrance and activities within the Marine Park are strictly controlled by Johor National Park Corporation, leading to a significant reduction of tourism activities in the area (Hassan 2013).

Coral species diversity surveys were conducted at Pulau Aur, Pulau Pemanggil, Pulau Besar, Pulau Sibu and Pulau Tinggi (Fig. 1) in 2012, when a total of 13 reefs were surveyed (depth range: 3–12 m) using 100 m transects perpendicular to the shoreline, for a total of 19 transects. Further surveys were conducted in 2016 for one reef at Pulau Tinggi using SCUBA timed swims (English et al. 1997), and seven reefs via transects perpendicular to the shoreline (Pulau Lima Kecil, Pulau Lima Besar, Pulau Tokong Rakit, Tokong Gantang, Tokong Chondong, Tokong Belalai and Tokong Chupak) (Fig. 1). The reefs surveyed in 2012 were all part of a larger marine protected area (MPA), whereas the reefs surveyed in 2016 were all outside the MPA (i.e., unprotected, non-MPA).

Corals were identified to species level based on distinct features of their morphological structure according to Veron (2000), using photographs and videos recorded during the survey. All identified species were standardized according to the World Register of Marine Species (Hoeksema and Cairns 2021) to account for synonyms and taxonomic change. The relative abundance and conservation status of each species were gathered from Veron (2000) and the IUCN Red List (IUCN 2019). Conservation categories that were used are Not Evaluated (NE), Data Deficient (DD), Least Concern (CC), Near Threatened (NT), Vulnerable (VU), Endangered (EN) and Critically Endangered (CR).

## ﻿Results

**Figure 2.**
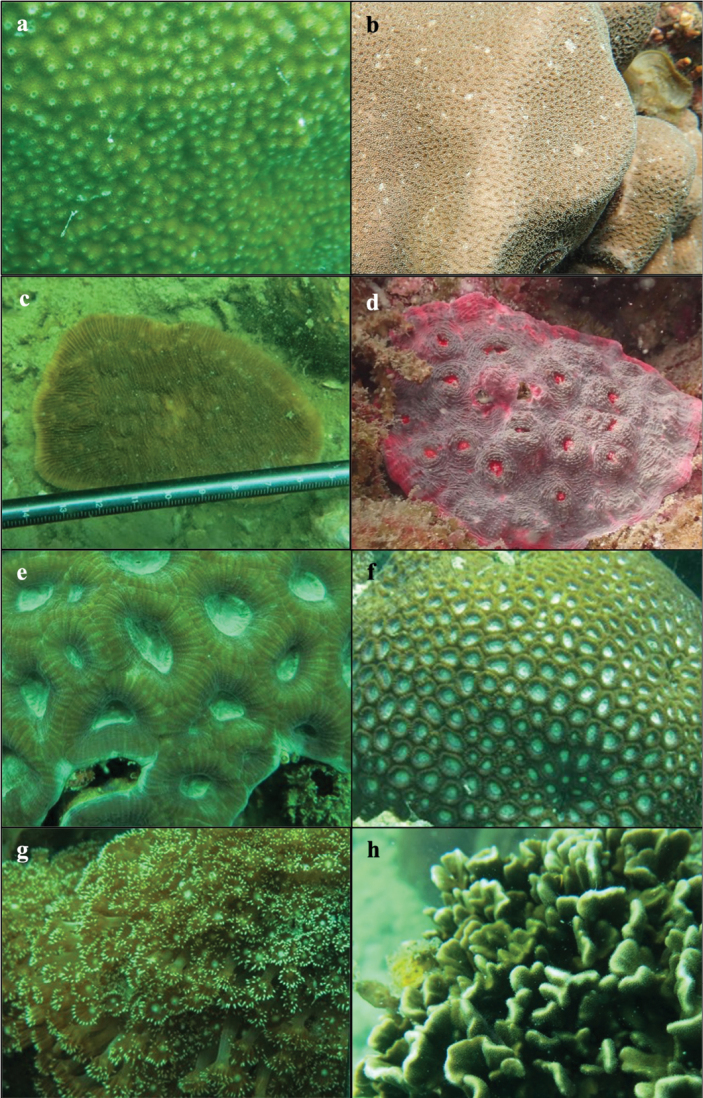
New records of scleractinian corals for the east coast of Peninsular Malaysia **a***Astreoporaexplanata***b***Coeloserismayeri***c***Halomitrapileus***d***Acanthastrearotundoflora***e***Favitesvasta***f***Paramontastraeaserageldini***g***Gonioporagracilis*, and **h***Pavonadivaricata*.

A total of 261 scleractinian coral species from 16 families and one *incertea sedis* were recorded during the 2012 (MPA; 243 species) and 2016 (non-MPA; 261 species) expeditions. Table 1 shows the checklist of scleractinian corals from Mersing Islands, with species arranged alphabetically by family and including records (155 species) from the previous survey published by Harborne et al. (2000). The current study found ten new records of scleractinian corals from the Mersing Islands in the larger Peninsular Malaysia east coast area (Fig. 1), i.e. *Acroporapectinata* (Brook, 1892); *Astreoporaexplanata* Veron, 1985; *Coeloserismayeri* Vaughan, 1918; *Halomitrapileus* (Linnaeus, 1758); *Acanthastrearotundoflora* Chevalier, 1975; *Favitesvasta* (Klunzinger, 1879); *Paramontastraeaserageldini* (Veron, 2000); *Seriatoporahystrix* Dana, 1846; *Gonioporagracilis* (Milne Edwards & Haime, 1849); and *Pavonadivaricata* Lamarck, 1816 (Fig. 2).

**Table 1.** Checklist of scleractinian corals from the Mersing Islands according to (a) Harborne et al. (2000); (b) Marine Park Biodiversity Expedition 2012; and (c) Johor 8 Islands Expedition 2016. Species denoted with an asterisk (*) are those considered to represent new records for the east coast of Peninsular Malaysia.

Order Scleractinia (17)	a	b	c	Abundance (sensu Veron, 2000)	IUCN Status
Family Acroporidae (79)					
*Acroporaabrotanoides* (Lamarck, 1816)		/		Sometimes common	LC
*Acroporaanthocercis* (Brook, 1893)			/	Sometimes common	VU
*Acroporaaspera* (Dana, 1846)	/	/		Sometimes common	VU
*Acroporaaustera* (Dana, 1846)		/		Usually uncommon	NT
*Acroporacerealis* (Dana, 1846)	/		/	Common	LC
*Acroporaclathrata* (Brook, 1891)		/	/	Common	LC
*Acroporacytherea* (Dana, 1846)	/	/	/	Common	LC
*Acroporadigitifera* (Dana, 1846)	/	/	/	Sometimes common	NT
*Acroporadivaricata* (Dana, 1846)	/	/	/	Common	NT
*Acroporaflorida* (Dana, 1846)	/	/	/	Common	NT
*Acroporagemmifera* (Brook, 1892)	/	/		Common	LC
*Acroporaglobiceps* (Dana, 1846)		/		Common	VU
*Acroporagrandis* (Brook, 1892)		/		Common	LC
*Acroporahemprichii* (Ehrenberg, 1834)		/		Common	VU
*Acroporahoeksemai* Wallace, 1997	/	/	/	Common	VU
*Acroporahorrida* (Dana, 1846)	/	/		Uncommon	VU
*Acroporahumilis* (Dana, 1846)	/	/		Common	NT
*Acroporahyacinthus* (Dana, 1846)	/	/	/	Common	NT
*Acroporaintermedia* (Brook, 1891)			/	Unknown	NE
*Acroporalatistella* (Brook, 1892)	/	/	/	Common	LC
*Acroporaloripes* (Brook, 1892)	/	/		Common	NT
*Acroporamicrophthalma* (Verrill, 1869)	/	/		Common	LC
*Acroporamillepora* (Ehrenberg, 1834)	/	/	/	Common	NT
*Acroporamonticulosa* (Brüggemann, 1879)	/	/		Uncommon	NT
*Acroporamuricata* (Linnaeus, 1758)	/	/	/	Common	NT
*Acroporanasuta* (Dana, 1846)	/	/		Common	NT
*Acroporapectinata Veron, 2000		/		Uncommon	DD
*Acroporarobusta* (Dana, 1846)	/	/		Common	LC
*Acroporasamoensis* (Brook, 1891)	/	/		Usually uncommon	LC
*Acroporasarmentosa* (Brook, 1892)	/	/		Common	LC
*Acroporasecale* (Studer, 1878)	/			Common	NT
*Acroporaselago* (Studer, 1879)	/		/	Sometimes common	NT
*Acroporasolitaryensis* Veron & Wallace, 1984	/	/		Rare	VU
*Acroporasubulata* (Dana, 1846)		/		Common	LC
*Acroporatenuis* (Dana, 1846)	/			Common	NT
*Acroporavalenciennesi* (Milne Edwards, 1860)		/		Common	LC
*Acroporavalida* (Dana, 1846)	/			Sometimes common	LC
*Acroporavaughani* Wells, 1954		/		Uncommon	VU
*Acroporayongei* Veron & Wallace, 1984	/	/		Common	LC
*Alveoporadaedalea* (Forskål, 1775)		/		Uncommon	VU
*Alveoporaspongiosa* Dana, 1846			/	Usually uncommon	NT
*Anacroporaforbesi* Ridley, 1884		/		Uncommon	LC
*Anacroporamatthaii* Pillai, 1973	/			Rare	VU
*Astreoporaexplanata Veron, 1985			/	Sometimes common	NE
*Astreoporagracilis* Bernard, 1896			/	Sometimes common	LC
*Astreoporalisteri* Bernard, 1896		/		Usually uncommon	LC
*Astreoporamyriophthalma* (Lamarck, 1816)	/	/		Common	LC
*Astreoporaocellata* Bernard, 1896		/		Usually rare	LC
*Isoporabrueggemanni* (Brook, 1893)	/	/		Common	VU
*Isoporacuneata* (Dana, 1846)		/		Common	VU
*Isoporapalifera* (Lamarck, 1816)	/	/		Common	NT
*Montiporaaequituberculata* Bernard, 1897	/	/	/	Common	LC
*Montiporacactus* Bernard, 1897		/		Common	VU
*Montiporacaliculata* (Dana, 1846)		/		Uncommon	VU
*Montiporacebuensis* Nemenzo, 1976	/	/		Uncommon	VU
*Montiporaconfusa* Nemenzo, 1967		/		Uncommon	NT
*Montiporadanae* Milne Edwards & Haime, 1851		/		Common	LC
*Montiporadelicatula* Veron, 2000		/		Uncommon	VU
*Montiporadigitata* (Dana, 1846)		/		Common	LC
*Montiporaeffusa* (Dana, 1846)		/		Uncommon	NT
*Montiporaflorida* Nemenzo, 1967		/		Common	VU
*Montiporafoliosa* (Pallas, 1766)		/		Common	NT
*Montiporafoveolata* (Dana, 1846)		/		Seldom common	NT
*Montiporagaimardi* Bernard, 1897	/	/		Sometimes common	VU
*Montiporahispida* (Dana, 1846)	/	/	/	Usually uncommon	LC
*Montiporainformis* Bernard, 1897		/		Common	LC
*Montiporamalampaya* Nemenzo, 1967	/			Common	VU
*Montiporamollis* Bernard, 1897	/	/		Common	LC
*Montiporamonasteriata* (Forskål, 1775)		/		Common	LC
*Montiporanodosa* (Dana, 1846)		/		Usually uncommon	NT
*Montiporapalawanensis* Veron, 2000		/		Uncommon	NT
*Montiporapeltiformis* Bernard, 1897		/		Uncommon	NT
*Montiporastellata* Bernard, 1897		/		Common	LC
*Montiporatuberculosa* (Lamarck, 1816)	/	/		Common	LC
*Montiporaturgescens* Bernard, 1897			/	Common	LC
*Montiporaturtlensis* Veron & Wallace, 1984		/		Common	VU
*Montiporavenosa* (Ehrenberg, 1834)		/		Uncommon	NT
*Montiporaverrucosa* (Lamarck, 1816)		/		Sometimes common	LC
*Montiporaverruculosa* Veron, 2000		/		Uncommon	VU
Famili Agariciidae (15)					
*Coeloserismayeri Vaughan, 1918		/	/	Uncommon	LC
*Gardineroserisplanulata* (Dana, 1846)	/	/		Usually uncommon	LC
*Leptoserisexplanata* Yabe & Sugiyama, 1941	/	/		Uncommon	LC
*Leptoserisfoliosa* Dinesen, 1980		/		Uncommon	LC
*Leptoserishawaiiensis* Vaughan, 1907		/		Uncommon	LC
*Leptoserismycetoseroides* Wells, 1954	/		/	Sometimes common	LC
*Leptoserisscabra* Vaughan, 1907		/		Usually uncommon	LC
*Pavonabipartita* Nemenzo, 1979	/			Uncommon	VU
*Pavonacactus* (Forskål, 1775)	/	/		Common	VU
*Pavonaclavus* Dana, 1846			/	Common	LC
*Pavonadanai* (Milne Edwards, 1860)		/		Uncommon	VU
*Pavonadecussata* (Dana, 1846)	/	/	/	Common	VU
*Pavonadivaricata Lamarck, 1816			/	Unknown	NE
*Pavonaexplanulata* (Lamarck, 1816)	/	/	/	Common	LC
*Pavonavarians* Verrill, 1864	/			Common	LC
Famili Astrocoeniidae (3)					
*Palauastrearamosa* Yabe & Sugiyama, 1941		/		Common	NT
*Stylocoeniellaarmata* (Ehrenberg, 1834)		/		Rare	LC
*Stylocoeniellaguentheri* (Bassett-Smith, 1890)	/	/		Uncommon	LC
Famili Dendrophylliidae (10)					
*Duncanopsammiapeltata* (Esper, 1790)	/	/	/	Common	VU
*Tubastraeacoccinea* Lesson, 1830	/		/	Unknown	NE
*Tubastraeadiaphana* (Dana, 1846)	/			Unknown	NE
*Tubastraeafaulkneri* Wells, 1982			/	Unknown	NE
*Tubastraeamicranthus* (Ehrenberg, 1834)	/		/	Unknown	NE
*Turbinariafrondens* (Dana, 1846)			/	Common	LC
*Turbinariairregularis* Bernard, 1896	/			Common	LC
*Turbinariamesenterina* (Lamarck, 1816)	/	/	/	Common	VU
*Turbinariareniformis* Bernard, 1896		/	/	Sometimes common	VU
*Turbinariastellulata* (Lamarck, 1816)	/	/	/	Usually uncommon	VU
Famili Diploastreidae (1)					
*Diploastreaheliopora* (Lamarck, 1816)	/	/	/	Common	NT
Famili Euphylliidae (8)					
*Euphylliacristata* Chevalier, 1971		/		Uncommon	VU
*Euphylliaglabrescens* (Chamisso & Eysenhardt, 1821)	/		/	Uncommon	NT
*Euphylliaparaglabrescens* Veron, 1990		/		Rare	VU
*Fimbriaphylliaancora* (Veron & Pichon, 1980)	/	/	/	Seldom common	VU
*Fimbriaphylliadivisa* (Veron & Pichon, 1980)	/	/		Seldom common	NT
*Fimbriaphylliaparadivisa* (Veron, 1990)		/		Uncommon	VU
*Galaxeaastreata* (Lamarck, 1816)	/	/		Common	VU
*Galaxeafascicularis* (Linnaeus, 1767)	/	/	/	Uncommon	NT
Famili Fungiidae (20)					
*Ctenactiscrassa* (Dana, 1846)	/			Usually uncommon	LC
*Ctenactisechinata* (Pallas, 1766)	/	/	/	Common	LC
*Cycloserisexplanulata* (van der Horst, 1922)	/	/		Uncommon	LC
*Cycloserisvaughani* (Boschma, 1923)		/		Rare	LC
*Danafungiahorrida* (Dana, 1846)	/			Uncommon	NE
*Danafungiascruposa* (Klunzinger, 1879)	/			Uncommon	LC
*Fungiafungites* (Linnaeus, 1758)	/	/	/	Common	NT
*Halomitrapileus (Linnaeus, 1758)		/	/	Usually uncommon	LC
*Heliofungiaactiniformis* (Quoy & Gaimard, 1833)		/		Common	VU
*Herpolithalimax* (Esper, 1792)	/	/		Common	LC
*Lithophyllonconcinna* (Verrill, 1864)	/	/		Common	LC
*Lithophyllonrepanda* (Dana, 1846)		/		Common	LC
*Lithophyllonundulatum* Rehberg, 1892	/	/	/	Usually uncommon	NT
*Lobactisscutaria* (Lamarck, 1801)		/		Common	LC
*Pleuractisgranulosa* (Klunzinger, 1879)	/			Usually uncommon	LC
*Pleuractismoluccensis* (Van der Horst, 1919)	/			Usually uncommon	LC
*Pleuractispaumotensis* (Stutchbury, 1833)	/	/		Common	LC
*Podabaciacrustacea* (Pallas, 1766)	/	/	/	Usually uncommon	LC
*Polyphylliatalpina* (Lamarck, 1801)	/	/	/	Common	LC
*Sandalolitharobusta* (Quelch, 1886)	/	/		Common	LC
Famili Lobophylliidae (23)					
*Acanthastreaechinata* (Dana, 1846)	/	/	/	Usually uncommon	LC
*Acanthastreahemprichii* (Ehrenberg, 1834)	/	/	/	Uncommon	VU
*Acanthastreapachysepta* (Chevalier, 1975)		/		Usually uncommon	NT
*Acanthastrearotundoflora Chevalier, 1975			/	Usually uncommon	NT
*Cynarinalacrymalis* (Milne Edwards & Haime, 1848)		/		Seldom common	NT
*Echinophylliaaspera* (Ellis & Solander, 1786)	/	/	/	Rare	LC
*Echinophylliaglabra* (Nemenzo, 1959)		/		Common	LC
*Homophylliaaustralis* (Milne Edwards & Haime, 1848)	/	/		Uncommon	LC
*Lobophylliaagaricia* (Milne Edwards & Haime, 1849)	/	/	/	Uncommon	LC
*Lobophylliacorymbosa* (Forskål, 1775)	/		/	Sometimes common	LC
*Lobophylliadiminuta* Veron, 1985		/		Uncommon	VU
*Lobophylliaflabelliformis* Veron, 2000		/	/	Usually uncommon	VU
*Lobophylliahataii* Yabe, Sugiyama & Eguchi, 1936		/	/	Uncommon	LC
*Lobophylliahemprichii* (Ehrenberg, 1834)	/	/	/	Common	LC
*Lobophylliaradians* (Milne Edwards & Haime, 1849)	/	/	/	Common	LC
*Lobophylliarecta* (Dana, 1846)	/	/	/	Common	LC
*Lobophylliarobusta* Yabe & Sugiyama, 1936	/			Uncommon	LC
*Lobophylliavalenciennesii* (Milne Edwards & Haime, 1849)		/		Uncommon	LC
*Lobophylliavitiensis* (Brüggemann, 1877)	/	/		Usually uncommon	NT
*Micromussalordhowensis* (Veron & Pichon, 1982)	/	/	/	Sometimes common	NT
*Oxyporacrassispinosa* Nemenzo, 1979		/		Uncommon	LC
*Oxyporaechinata* (Saville Kent, 1871)		/	/	Usually rare	LC
*Oxyporalacera* (Verrill, 1864)	/		/	Common	LC
Famili Merulinidae (57)					
*Astraeosmiliatumida* (Matthai, 1928)	/	/		Uncommon	NT
*Astreacurta* Dana, 1846	/		/	Common	LC
*Coelastreaaspera* (Verrill, 1866)		/	/	Common	LC
*Cyphastreamicrophthalma* (Lamarck, 1816)		/	/	Common	LC
*Cyphastreaocellina* (Dana, 1846)		/		Rare	VU
*Cyphastreaserailia* (Forskål, 1775)			/	Common	LC
*Dipsastraeaamicorum* (Milne Edwards & Haime, 1849)	/			Uncommon	LC
*Dipsastraeafavus* (Forskål, 1775)		/	/	Common	LC
*Dipsastraeahelianthoides* (Wells, 1954)			/	Sometimes common	NT
*Dipsastraeamaritima* (Nemenzo, 1971)	/			Uncommon	NT
*Dipsastraeapallida* (Dana, 1846)	/			Less common	LC
*Dipsastraeaspeciosa* (Dana, 1846)			/	Common	LC
*Dipsastraeaveroni* (Moll & Best, 1984)		/	/	Rare	NT
*Echinoporagemmacea* (Lamarck, 1816)	/		/	Usually uncommon	LC
*Echinoporahorrida* Dana, 1846		/	/	Uncommon	NT
*Echinoporalamellosa* (Esper, 1791)		/	/	Common	LC
*Echinoporamammiformis* (Nemenzo, 1959)		/	/	Common	NT
*Echinoporapacifica* Veron, 1990	/	/	/	Usually uncommon	NT
*Favitesabdita* (Ellis & Solander, 1786)	/	/	/	Common	NT
*Favitescomplanata* (Ehrenberg, 1834)		/		Sometimes common	NT
*Favitesflexuosa* (Dana, 1846)		/	/	Sometimes common	NT
*Faviteshalicora* (Ehrenberg, 1834)	/	/	/	Usually uncommon	NT
*Favitesmagnistellata* (Milne Edwards & Haime, 1849)	/		/	Usually uncommon	NT
*Favitesmelicerum* (Ehrenberg, 1834)		/		Rare	NT
*Favitespentagona* (Esper, 1790)		/	/	Sometimes common	LC
*Favitesvalenciennesii* (Milne Edwards & Haime, 1849)		/		Usually uncommon	NT
*Favitesvasta (Klunzinger, 1879)			/	Uncommon	NT
*Goniastreaedwardsi* Chevalier, 1971	/	/		Common	LC
*Goniastreafavulus* (Dana, 1846)	/	/	/	Uncommon	NT
*Goniastreapectinata* (Ehrenberg, 1834)	/	/	/	Common	LC
*Goniastrearetiformis* (Lamarck, 1816)		/	/	Common	LC
*Goniastreastelligera* (Dana, 1846)	/	/	/	Common	NT
*Hydnophoraexesa* (Pallas, 1766)	/	/	/	Common	NT
*Hydnophoragrandis* Gardiner, 1904	/	/		Usually uncommon	LC
*Hydnophoramicroconos* (Lamarck, 1816)	/	/	/	Uncommon	NT
*Hydnophorarigida* (Dana, 1846)		/	/	Sometimes common	LC
*Leptoriaphrygia* (Ellis & Solander, 1786)	/	/	/	Common	NT
*Merulinaampliata* (Ellis & Solander, 1786)	/	/	/	Usually common	LC
*Merulinacylindrica* (Milne Edwards & Haime, 1849)	/		/	Uncommon	LC
*Merulinascabricula* Dana, 1846	/	/		Common	LC
*Mycediumelephantotus* (Pallas, 1766)	/	/	/	Common	LC
*Orbicellaannularis* (Ellis & Solander, 1786)		/		Rare	NE
*Oulophylliabennettae* (Veron, Pichon & Wijsman-Best, 1977)	/	/		Uncommon	NT
*Oulophylliacrispa* (Lamarck, 1816)	/	/	/	Uncommon	NT
*Paramontastraeasalebrosa* (Nemenzo, 1959)		/		Rare	VU
*Paramontastraeaserageldini (Veron, 2000)			/	Rare	VU
*Pectiniaalcicornis* (Saville Kent, 1871)		/	/	Usually uncommon	VU
*Pectinialactuca* (Pallas, 1766)		/		Common	VU
*Pectiniamaxima* (Moll & Best, 1984)		/		Uncommon	EN
*Pectiniapaeonia* (Dana, 1846)	/	/	/	Common	NT
*Platygyraacuta* Veron, 2000		/		Sometimes common	NT
*Platygyradaedalea* (Ellis & Solander, 1786)	/		/	Common	LC
*Platygyralamellina* (Ehrenberg, 1834)	/	/	/	Usually uncommon	NT
*Platygyrapini* Chevalier, 1975		/	/	Usually uncommon	LC
*Platygyrasinensis* (Milne Edwards & Haime, 1849)	/	/	/	Usually uncommon	LC
*Platygyraverweyi* Wijsman-Best, 1976		/		Usually uncommon	NT
*Trachyphylliageoffroyi* (Audouin, 1826)		/		Rare	NT
Famili Plerogyridae (2)					
*Physogyralichtensteini* (Milne Edwards & Haime, 1851)		/	/	Common	VU
*Plerogyrasinuosa* (Dana, 1846)	/	/	/	Usually uncommon	NT
Famili Plesiastreidae (1)					
*Plesiastreaversipora* (Lamarck, 1816)	/	/		Unknown	LC
Famili Pocilloporidae (7)					
*Pocilloporadamicornis* (Linnaeus, 1758)	/	/	/	Common	LC
*Pocilloporagrandis* Dana, 1846		/		Common	NT
*Pocilloporameandrina* Dana, 1846		/		*Common	LC
*Pocilloporaverrucosa* (Ellis & Solander, 1786)	/	/		Common	LC
*Seriatoporahystrix Dana, 1846		/		Common	LC
*Stylophorapistillata* (Esper, 1792)		/		Common	NT
*Stylophorasubseriata* (Ehrenberg, 1834)	/	/		Common	LC
Famili Poritidae (21)					
*Gonioporacolumna* Dana, 1846		/	/	Common	NT
*Gonioporadjiboutiensis* Vaughan, 1907			/	Common	LC
*Gonioporagracilis (Milne Edwards & Haime, 1849)			/	Unknown	NE
*Gonioporalobata* Milne Edwards, 1860		/	/	Common	NT
*Gonioporanorfolkensis* Veron & Pichon, 1982		/		Uncommon	LC
*Gonioporaplanulata* (Ehrenberg, 1834)		/		Usually uncommon	VU
*Gonioporastokesi* Milne Edwards & Haime, 1851		/		Uncommon	NT
*Poritesannae* Crossland, 1952	/	/	/	Common	NT
*Poritesattenuata* Nemenzo, 1955		/		Common	VU
*Poritesaustraliensis* Vaughan, 1918		/		common	LC
*Poritescylindrica* Dana, 1846	/	/		Common	NT
*Poritesdensa* Vaughan, 1918		/		Sometimes common	NT
*Poritesevermanni* Vaughan, 1907	/	/	/	Usually uncommon	DD
*Poriteslatistellata* Quelch, 1886		/		Uncommon	LC
*Poriteslichen* (Dana, 1846)		/		Common	LC
*Poriteslobata* Dana, 1846		/	/	Common	NT
*Poriteslutea* Milne Edwards & Haime, 1851		/	/	Common	LC
*Poritesmonticulosa* Dana, 1846		/		Common	LC
*Poritesnigrescens* Dana, 1846		/		Sometimes common	VU
*Poritesrus* (Forskål, 1775)	/	/		Common	LC
*Poritessolida* (Forskål, 1775)	/	/	/	Common	LC
Famili Psammocoridae (6)					
*Psammocoracolumna* Dana, 1846	/	/	/	Sometimes common	LC
*Psammocoracontigua* (Esper, 1794)	/	/		Common	NT
*Psammocoradigitata* Milne Edwards & Haime, 1851	/	/	/	Usually uncommon	NT
*Psammocoraexesa* Dana, 1846	/	/		Common	LC
*Psammocorahaimiana* Milne Edwards & Haime, 1851		/		Uncommon	LC
*Psammocoraprofundacella* Gardiner, 1898	/			Uncommon	LC
Famili Rhizangiidae (1)					
*Pseudosiderastreatayamai* Yabe & Sugiyama, 1935	/			Uncommon	NT
Famili Leptastreidae (3)					
*Leptastreaaequalis* Veron, 2000		/		Rare	VU
*Leptastreapurpurea* (Dana, 1846)	/	/	/	Common	LC
*Leptastreatransversa* Klunzinger, 1879		/		Uncommon	LC
Famili Scleractinia incertae sedis (4)					
*Pachyserisfoliosa* Veron, 1990		/		Uncommon	LC
*Pachyserisgemmae* Nemenzo, 1955		/	/	Rare	NT
*Pachyserisrugosa* (Lamarck, 1801)	/	/		Common	VU
*Pachyserisspeciosa* (Dana, 1846)	/	/	/	Common	LC

Of the 16 families recorded, Acroporidae was the richest with a total of 79 species: 39 *Acropora* species, 28 *Montipora* species and four from other genera (Table 1). Six per cent (16) of species from the list were considered ‘rare’ in abundance according to Veron (2000), whereby the species can be common in a specific area but rare overall. According to the IUCN Red List, many coral species we observed were classified as of Least Concern (46.7%), Near Threatened (29.5%) or Vulnerable (18.8%). Only one species, *Pectiniamaxima*, was categorised as Endangered (EN). The remaining species were Not Evaluated (3.8%) or classified as Data Deficient (0.8%).

## ﻿Discussions and conclusions

The current study provides an updated species checklist of scleractinian corals from coral reefs around the Mersing Islands. A total of 261 scleractinian species were recorded, including ten new records for the east coast of Peninsular Malaysia, from where 398 species were previously reported (Huang et al. 2015). Compared to previous findings by Harborne et al. (2000) (155 species recorded from a subset of reefs around the Mersing Islands), we find the coral diversity around the Mersing Islands to be comparable, if not slightly higher, than other reefs in the region, i.e., Pulau Tioman with 239 species (Akmal et al. 2019) (i.e., north of the Mersing Islands) and Singapore with 255 species (Huang et al. 2009) (i.e., south of the Mersing Islands). The South China Sea in the Central Indo Pacific holds a high biodiversity of scleractinian corals, with a total recorded number of 571 species. The diversity found around the Mersing Islands represents ~ 45% of the total recorded coral fauna of the South China Sea and ~65% of the total recorded fauna from the east coast of Peninsular Malaysia. Previous records and records from the current study account for a total of 413 scleractinian coral species for reefs along the east coast of Peninsular Malaysia. These include eight new records of coral species at Pulau Tioman and Pulau Redang by Akmal et al. (2019) and the ten (10) new records from this study.

The ten new records of coral species for the east coast of Peninsular Malaysia found during this study are known to be widely distributed in the Indo-West Pacific Ocean (east coast of Africa to Japan and Melanesia) (Veron 2000; Cairns and Hoeksema 2022; GBIF 2022). Two of these species (*Acanthastrearotundoflora* and *Seriatoporahystrix*) had previously been reported from Singapore’s southern islands (Huang et al. 2009), whereas another species (*Pavonadivaricata*) was previously recorded from the west coast of Peninsular Malaysia (Affendi and Rosman 2011). However, we note that all the newly recorded coral species found were rarely observed in our surveys, suggesting that their occurrence along the east coast of Peninsular Malaysia may be relatively low. Given the vastness of the coral reef area around the Mersing Islands and the complexity of reef ecosystems, together with seagrass meadows, such as those at Pulau Tinggi (Ooi et al. 2011) and Pulau Besar (Lee et al. 2010), we posit that the current account of coral diversity in this region may yet be underestimated. Further surveys around the Mersing Islands are likely to yield new findings, as visual surveys have only been conducted once at each study reef site. Although hard scleractinian corals form the basis of coral reef ecosystems, information about other reef-related species’ diversity and abundance is also crucial for marine area planning (e.g., determining management strategies and protection status). Based on the results of the current study, we propose that more surveys should be conducted around the Mersing Islands, extending investigations to other taxa where possible.

Biodiversity and taxonomic studies on the scleractinian corals of Peninsular Malaysia are in their infancy compared to neighbouring regions, e.g., Singapore (Huang et al. 2009) and Sabah, East Malaysia (Waheed and Hoeksema 2013, 2014; Waheed et al. 2015). Given recent findings around the region, such as the new genus and species records of *Micromussaanalusensis* by Ng et al. (2019), the increased occurrence and records of *Pocilloporaacuta* (Poquita-Du et al. 2017; Torres and Ravago-Gotanco 2018), and the cryptic speciation in *Pachyserisspeciosa* (Bongaerts et al. 2021; Feldman et al. 2021), we can expect important scleractinian discoveries for the Mersing Islands (and other coral reefs in Malaysia) should we aim to further explore and examine these underexplored reefs.

## References

[B1] Affendi YA Rosman FR (2011) Current knowledge on scleractinian coral diveristy of Peninsular Malaysia. In: Kamarrudin I Mohamed CAR Rozaimi MJ Kee Alfian BAA Fitra AZ Lee JN (Eds) Malaysia’s Marine Biodiversity: inventory and current status.Department of Marine Park Malaysia, Putrajaya, 21–31.

[B2] Akmal KF Shahbudin S Faiz MHM Hamizan YM (2019) Diversity and abundance of scleractinian corals in the East Coast of Peninsular Malaysia: A case study of Redang and Tioman Islands. Ocean Science Journal 54(3): 435–456. 10.1007/s12601-019-0018-6

[B3] Bongaerts P Cooke IR Ying H Wels D den Haan S Hernandez-Agreda A Brunner CA Dove S Englebert N Eyal G Forêt S Grinblat M Hay KB Harii S Hayward DC Lin Y Mihaljević M Moya A Muir P Sinniger F Smallhorn-West P Torda G Ragan MA van Oppen MJH Hoegh-Guldberg O (2021) Morphological stasis masks ecologically divergent coral species on tropical reefs. Current Biology 31(11): 2286–2298. [e8] 10.1016/j.cub.2021.03.028 33811819

[B4] Cairns S Hoeksema B (2022) World List of Scleractinia. In: Bánki O, Roskov Y, Döring M, Ower G, Vandepitte L, Hobern D, Remsen D, Schalk P, DeWalt RE, Keping M, Miller J, Orrell T, Aalbu R, Adlard R, Adriaenssens EM, Aedo C, Aescht E, Akkari N, Alfenas-Zerbini P, et al. (Eds) Catalogue of Life Checklist (ver. (03/2022)). 10.48580/dfpd-3g9

[B5] Chong VC Lee PKY Lau CM (2010) Diversity, extinction risk and conservation of Malaysian fishes. Journal of Fish Biology 76(9): 2009–2066. 10.1111/j.1095-8649.2010.02685.x 20557654

[B6] English S Wilkinson C Baker V (1997) Survey Manual for Tropical Marine Resources. Australia Institute of Marine Science, Australia, 390 pp.

[B7] Feldman B Afiq-Rosli L Simon-Blecher N Bollati E Wainwright BJ Bongaerts P Huang D Levy O (2021) Distinct lineages and population genomic structure of the coral *Pachyserisspeciosa* in the small equatorial reef system of Singapore. Coral Reefs. 10.1007/s00338-021-02160-4

[B8] GBIF (2022) Global Biodiversity Information. Facility occurrences, version 2 April 2022.

[B9] Harborne A Fenner D Barnes A Beger M Harding S Roxburgh T (2000) Status Report On The Coral Reefs Of The East Coast Of Peninsular Malaysia. Coral Cay Conservation Ltd, Malaysia, 88 pp.

[B10] Hassan H (2013) Taman Laut Sultan Iskandar: tarikan pelancongan, tumpuan penyelam skuba. http://www.utusan.com.my

[B11] Hoegh-Guldberg O Pendleton L Kaup A (2019) People and the changing nature of coral reefs. Regional Studies in Marine Science 30: e100699. 10.1016/j.rsma.2019.100699

[B12] Hoeksema BW Cairns S (2021) World List of Scleractinia. http://www.marinespecies.org/scleractinia [on 2021–01–20]

[B13] Huang D Tun KPP Chou LM Todd PA (2009) An inventory of zooxanthellate scleractinian corals in Singapore, including 33 new records. The Raffles Bulletin of Zoology 22: 69–80. 10.26107/RBZ-2020-0056

[B14] Huang D Licuanan WY Hoeksema BW Chen CA Ang PO Huang H Lane DJW Vo ST Waheed Z Affendi YA Yeemin T Chou LM (2015) Extraordinary diversity of reef corals in the South China Sea. Marine Biodiversity 45(2): 157–168. 10.1007/s12526-014-0236-1

[B15] Huang D Hoeksema BW Affendi YA Ang PO Chen CA Huang H Lane DJW Licuanan WY Vibol O Vo ST Yeemin T Chou LM (2016) Conservation of reef corals in the South China Sea based on species and evolutionary diversity. Biodiversity and Conservation 25(2): 331–344. 10.1007/s10531-016-1052-7

[B16] IUCN (2019) The IUCN Red List of Threatened Species Version 2019-3. https://www.iucnredlist.org [on 2019–11–13]

[B17] Lee JN Ahmad SB Badriah H Adzis KAA Rahman MAA Senik S Mohamed CAR (2010) Preliminary notes of seagrasses from Pulau Besar, Johor. In: Mohamed CAR Sahrani FK Manaf AA Omar M Cob AC Lee JN (Eds) The Studies of Johor East Coast: Preserve Mersing Heritage.Marine Ecosystem Research Centre, UKM, Bangi, Malaysia, 167–172.

[B18] Ministry of Natural Resources and Environment (2016) National Policy on Biological Diversity 2016-2025. Ministry of Natural Resources and Environment (NRE), Malaysia, 112 pp.

[B19] Ng CSL Jain SS Nguyen NTH Sam SQ Kikuzawa YP Chou LM Huang D (2019) New genus and species record of reef coral *Micromussaanalusensis* in the southern South China Sea. Marine Biodiversity Records 12(1): e17. 10.1186/s41200-019-0176-3

[B20] Ooi JLS Kendrik GA Van Niel KP Affendi YA (2011) Knowledge gaps in tropical Southeast Asian seagrass systems. Estuarine, Coastal and Shelf Science 92(1): 118–131. 10.1016/j.ecss.2010.12.021

[B21] Ponnampalam LS Fairul Izmal JH Adulyanukosol K Ooi JLS Reynolds JE III (2015) Aligning conservation and research priorities for proactive species and habitat management: The case of dugongs Dugong dugon in Johor, Malaysia. Oryx 49(4): 743–749. 10.1017/S0030605313001580

[B22] Poquita-Du RC Ng CSL Loo JB Afiq-Rosli L Tay YC Todd PA Chou LM Huang D (2017) New evidence shows that *Pocillopora* ‘*Pocilloporadamicornis*-like’ corals in Singapore are actually *Pocilloporaacuta* (Scleractinia: Pocilloporidae). Biodiversity Data Journal 5: e11407. 10.3897/BDJ.5.e11407 PMC5345112 28325983

[B23] Praveena SM Siraj SS Aris AZ (2012) Coral reefs studies and threats in Malaysia: A mini review. Reviews in Environmental Science and Biotechnology 11(1): 27–39. 10.1007/s11157-011-9261-8

[B24] Said MZ Komoo I Mohamad ET Ali CA Ahmad N Wahid MEA Rajimin MF (2021) Geological, biological, cultural and local wisdom heritage a key element of Mersing Geopark development. Bulletin of the Geological Society of Malaysia 71: 89–98. 10.7186/bgsm71202108

[B25] Toda T Okashita T Maekawa T Kee Alfian BAA Rajuddin MKM Nakajima R Chen W Takahashi KT Othman BHR Terazaki M (2007) Community structure of coral reefs around Peninsular Malaysia. Journal of Oceanography 63: 113–123. https://10.1007/s10872-007-0009-6

[B26] Tong PS (2020) More policies and laws, is it better for biodiversity conservation in Malaysia? Conservation Science and Practice 235(8): 1–11. 10.1111/csp2.235

[B27] Torres AF Ravago-Gotanco R (2018) Rarity of the “common” coral *Pocilloporadamicornis* in the western Philippine archipelago. Coral Reefs 37(4): 1209–1216. 10.1007/s00338-018-1729-3

[B28] UNEP (2007) National Reports on Coral Reefs in the Coastal Water of the South China Sea. UNEP/GEF?SCS Technical Publications No. 11, Thailand, 118 pp.

[B29] Veron JEN (2000) Corals of the world. Volume 1–3. Australian Institute of Marine Science, Townsville, Australia.

[B30] Veron JEN Stafford-Smith M De Vantier L Turak E (2015) Overview of distribution patterns of zooxanthellate Scleractinia. Frontiers in Marine Science 1: e81. 10.3389/fmars.2014.00081

[B31] Waheed Z Hoeksema BW (2013) A tale of two winds: Species richness patterns of reef corals around the Semporna peninsula, Malaysia. Marine Biodiversity 43(1): 37–51. 10.1007/s12526-012-0130-7

[B32] Waheed Z Hoeksema BW (2014) Diversity patterns of scleractinian corals at Kota Kinabalu, Malaysia, in relation to exposure and depth. The Raffles Bulletin of Zoology 62: 66–82.

[B33] Waheed Z van Mil HGJ Syed Hussein MA Jumin R Golam Ahad B Hoeksema BW (2015) Coral reefs at the northernmost tip of Borneo: An assessment of scleractinian species richness patterns and benthic reef assemblages. PLoS ONE 10(12): e0146006. 10.1371/journal.pone.0146006 PMC4697805 26719987

